# Bicarbonate-Hydrogen Peroxide System for Treating Dyeing Wastewater: Degradation of Organic Pollutants and Color Removal

**DOI:** 10.3390/toxics11040366

**Published:** 2023-04-11

**Authors:** Néstor A. Urbina-Suarez, Christian Rivera-Caicedo, Ángel Darío González-Delgado, Andrés F. Barajas-Solano, Fiderman Machuca-Martínez

**Affiliations:** 1 Department of Environmental Sciences, Universidad Francisco de Paula Santander, Av. Gran Colombia No. 12E-96, Cucuta 540003, Colombia; 2 School of Natural Resources and Environment, Universidad del Valle, Ciudad Universitaria Meléndez, Calle 13 # 100-00, Cali 760015, Colombia; 3 Biotechnological Engineering Program, Universidad Francisco de Paula Santander, Av. Gran Colombia No. 12E-96, Cucuta 540003, Colombia; 4 Nanomaterials and Computer Aided Process Engineering Research Group (NIPAC), Chemical Engineering Department, Faculty of Engineering, Universidad de Cartagena, Av. Del Consulado Calle 30 No. 48-152, Cartagena 130015, Colombia; 5 School of Chemical Engineering, Center of Excellence in New Materials (CENM), Universidad del Valle Ciudad Universitaria Meléndez, Calle 13 # 100-00, Cali 760015, Colombia

**Keywords:** advanced oxidation processes, dyes, oxidative degradation, bicarbonate, hydrogen peroxide

## Abstract

The textile industry is a global economic driving force; however, it is also one of the most polluting industries, with highly toxic effluents which are complex to treat due to the recalcitrant nature of some compounds present in these effluents. This research focuses on the removal of Chemical Oxygen Demand (COD), color, Total Organic Carbon (TOC), and Ammoniacal Nitrogen (N-NH_3_) on tannery wastewater treatment through an advanced oxidation process (AOPs) using sodium bicarbonate (NaHCO_3_), hydrogen peroxide (H_2_O_2_) and temperature using a central composite non-factorial design with a surface response using Statistica 7.0 software. All experiments used a 500 mL reactor with 300 mL of tannery wastewater from a company in Cúcuta, Colombia. The physicochemical characterization was done to determine the significant absorbance peaks about the color in the wavelengths between 297 and 669 nm. Statistical analysis found that the concentration of NaHCO_3_ affects the removal of color and N-NH_3_; however, it did not affect COD and TOC. The optimal process conditions for removing the different compounds under study were: NaHCO_3_ 1 M, H_2_O_2_ 2 M, and 60 °C, with efficiencies of 92.35%, 31.93%, 68.85%, and 35.5% N-NH_3_, COD, color, and TOC respectively. It can be concluded that AOPs using H_2_O_2_ and NaHCO_3_ are recommended to remove color and N-NH_3_.

## 1. Introduction

The textile industry is one of the largest in the world, uses more than 8000 chemical products in diverse textile manufacturing processes, and generates large amounts of wastewater [1]. The toxic potential of dyes and colorants was underestimated in comparison to other substances in water effluents until recent years [2]. Azo-type dyes are the most common substances in these effluents [3], along with other substances such as polyvinyl alcohol (PVA), carboxymethylcellulose, surfactants, organic processing acids, sulfur, formaldehyde, detergents, oil, dispersants, NaNO_3_, NaCl, and the salts of Na_2_SO_4_ [4]. The dyes used in these processes may contain heavy metals such as chromium, copper, and cobalt; chemicals such as caustic soda, sodium carbonate, and salts [5,6,7,8]; because of these characteristics, dyes and colorants are considered highly toxic to ecosystems, animals, and people [9] since dyes have been reported to have carcinogenic, allergic and dermal effects. These effects entail that the production and use of these compounds may not be safe, especially considering that almost 200 billion liters/year of colored effluents are generated [10].

Wastewater from the textile sector is difficult to treat due to its poorly degradable recalcitrant organic contaminants [11]. Traditional wastewater treatment methods are ineffective due to the non-biodegradability of the dyes; this has led to paying attention to AOPs for the degradation of non-biodegradable organic compounds. AOPs reduce the toxicity of the treated effluent and mineralize certain pollutants through oxidation reactions with a potent non-selective hydroxyl radical (OH^−^) by removing recalcitrant organic compounds and managing these waters. The above transform AOPs into promising alternatives for wastewater treatment processes [12,13,14,15,16]. Unlike conventional treatments, AOPs lead to reduced toxicity of the treated effluent [12,13].

Despite the efficiency of AOPs based on H_2_O_2,_ limitations still exist. Among them, the pH range is one of the main constraints [17], considering that in an acid medium, the results are good but not in alkaline, where secondary contamination may occur from metal catalysts [18]. Other disadvantages of AOPs are related to the relatively high costs for chemical reagents or energy use, and certain specific processes can generate a secondary contaminant [19]. Consequently, recent research aims to carry out combined methods or modifications to AOPs, to increase the method’s efficiency [12,20,21,22]. Bicarbonate-activated hydrogen peroxide (BAP) has received wide attention in dye degradation [23]. It has been reported that using bicarbonate to activate hydrogen peroxide allows for working at higher pH ranges, and higher concentrations of H_2_O_2_ and carbonate could remove dyes from wastewater efficiently [24]. The reaction between H_2_O_2_ and HCO_3_ could produce the formation of reactive species such as peroxymonocarbonate (HCO_4_^−^), singlet oxygen (O_2_), hydroxide (OH), and carbonate (CO_3_^−^), which can degrade organic compounds [20]. The hydrogen peroxide-bicarbonate system can create environmentally friendly oxidation technologies as alternatives to current AOPs [25].

Most studies have investigated the activation of H_2_O_2_ with HCO_3_ for its use in synthetic wastewater; there is no evidence of using this system in real wastewater from the textile industry. Therefore, the present investigation evaluated an advanced oxidation process using H_2_O_2_/HCO_3_ to treat real wastewater from a tannery company from Norte de Santander (Colombia). The water was characterized to determine the polluting organic load, and then H_2_O_2_/HCO_3_ advanced oxidation method was evaluated to establish the removal efficiency of COD, Color, N-NH_3_, and TOC. The effect of temperature and H_2_O_2_ and NaHCO_3_ concentration was determined using an experimental design to find optimal operating conditions.

## 2. Materials and Methods

### 2.1. Wastewater Collection

The wastewater samples were collected in a textile factory in Cúcuta (Colombia). 5 gallons were collected, and cold storage was used for subsequent physicochemical analysis. The physicochemical characterization is carried out according to Standard Methods Edition 23 [26]; The parameters analyzed are shown in Table 1. 

### 2.2. Experimental Design

A central Composite non-factorial 3^3^ design with a response surface using Statistica 7.0 software was implemented. The effect of temperature, hydrogen peroxide concentration, and bicarbonate concentration on the removal of color, N-NH_3_, COD, and TOC was evaluated to obtain 20 experiments that were analyzed in triplicate. The experimental design is shown in Table 2.

The experiments were carried out in 500 mL reactors using a working volume of 300 mL of residual dyeing water; the temperature and agitation were controlled using the agitation plate, where these factors were adjustable, and the temperature was estimated utilizing a thermocouple coupled to the stir plate. The dyeing water was decanted in 1 L cones to remove solids. The reaction time of the process was one h. The data obtained were analyzed using the Statistica 7.0 Software to get response surface and Pareto graphs. The equations of each variable with the optimal conditions of the process for removal of COD, TOC, color, and N-NH_3_ were subsequently established.

### 2.3. Analytical Methods

#### 2.3.1. COD Determination

The quantification of the COD was determined by the method 5220 C—Closed reflux of the standard methods edition 23 [27], using potassium dichromate as an oxidant agent in an acid medium. For this, its digestion was carried out by adding 1.5 mL of digester solution and 3.5 mL of catalyst solution to 2.5 mL of sample; it was stirred in the vortex, heated for 2 h at 150 °C, and let cool to room temperature. Subsequently, absorbance measurements were made in a TERMO GENESYS UV-Visible spectrophotometer at a wavelength of 610 nm, and COD was determined. The COD calibration curve was generated from 5 patterns of potassium acid phthalate, made up of 5 concentrations 0, 50, 100, 250, and 500 mg L^−1^. Each experiment was carried out by duplicate.

#### 2.3.2. TOC Determination

For the quantification of TOC, A TORCH TOC analyzer from TELEDYNE, Cincinnati, USA, was used. The operating conditions were sample volume of 0.5 mL, water chase volume of 1.0 mL, injection line rinse on, injection line rinse volume of 0.5 mL, acid volume of 0.5 mL, ICS parge flow 200 mL min^−1^, carrier gas delay time of 0.40 min, ICS parge time 50 min, detector sweep flow 500 mL min^−1^, furnace sweep time 1.0 min and system flow 200 mL min^−1^.

#### 2.3.3. Determination of N-NH_3_

It used the 4500-NH_3_ F Phenate method. For this, 1 mL of phenol solution, 1 mL of sodium nitroprusside solution, and 2.5 mL of oxidizing solution were added to the sample, mixing well after each addition. The samples were covered with a paraffin film to ensure color development. Samples were left at room temperature (22–27 °C) in dim light for one h to allow the color to develop. The color is stable for 24 h. Absorbance at 640 nm was measured in a Model DR3900 UV-VIS Spectrophotometer, HACH, Ames, LA, USA. The calibration curve was made using a mother solution of ammonium chloride, which was obtained by dissolving 3.819 g of NH_4_Cl anhydrous (dried a 100 °C) in 1000 mL (1.00 mL = 1.00 mg N = 1.22 mg NH_3_). The calibration curve was designed for 0–50 mg L^−1^.

#### 2.3.4. Determination of Color

To determine color, a scan was performed in a GENESYS spectrophotometer, THERMO, Massachusetts, USA. The scan was performed on each sample starting from a wavelength of 250 to 750 nm at a temperature of 25 °C; the behavior of the curve determined the absorbance peaks. The interval or separation of the measurements was calibrated in “1 nm”. To measure the baseline of the spectra, deionized water was used, and later, to run the analyses, the standard and post-treatment problem samples were used.

## 3. Results and Discussion

### 3.1. Physicochemical Characterization

The results of the physicochemical characterization of wastewater from the dye industry and the comparison with research done by other authors are shown in Table 3. The recollected wastewater from the dye industry has many pollutants, such as chemical compounds and solids. Very high values were reported for COD (598 mg L^−1^), chloride (850.67 mg L^−1^), total solids (1367 mg L^−1^), and sulfates (385 mg L^−1^). The wastewaters from textile industries have an intense color due to the chemical dyes used in the dyeing process, a large number of suspended solids (SS), fluctuating pH (4–9), and a high COD concentration [27,28,29,30,31,32,33]. However, the composition and concentration of these compounds are highly variable for textile wastewater, depending on the manufacturing processes, type of fibers, amount of water used in the process, and chemical products [27,28], parameters like turbidity, color, COD, SS, conductivity, pH, salinity and chemical elements, may fluctuate accordingly with the part of the process or installation [34]. This could be explained since weaving, assembly, dyeing, washing, ironing, and finishing are the most common steps in the manufacturing process, the dyeing process where dyes are used which generate a large part of the contaminant load [32]. The most abundant dyes are the azo-type, which is characterized by the existence of at least an azo bond R-N = N − R’, in which R and R’ can be aryl or alkyl and which has an aromatic ring, which generates the presence of nitrogenous compounds in the water [29]. Some textile wastewater can already have bicarbonate loads between 541 ± 5.50 mg L^−1^ [30]. When comparing the results of Table 3 with the Colombian standard for liquid discharges, Resolution 0631 of 2015 [35], it was determined that the samples of this study exceed the maximum permitted level of discharge for the manufacture of textile products, where for COD the permissible limit is from 400 mg L^−1^ O_2_, total suspended solids (TSS) of 50 mg L^−1^ and chloride 1200 mg L^−1^. Therefore, these samples harm human health and the environment’s well-being.

### 3.2. Removal of COD, TOC, Color, and N-NH_3_

**Figure 1:**
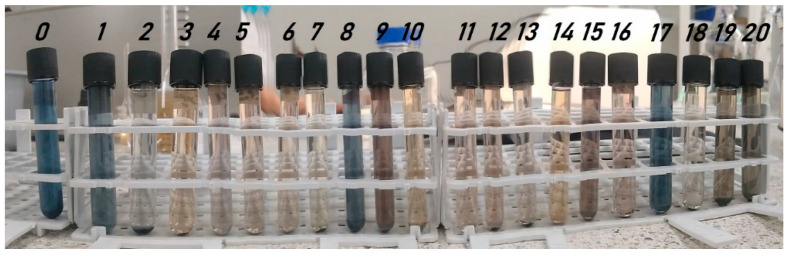
Dyeing wastewater samples before (0) and after treatment (1–20) (0–20 from left to right).

**Figure 2:**
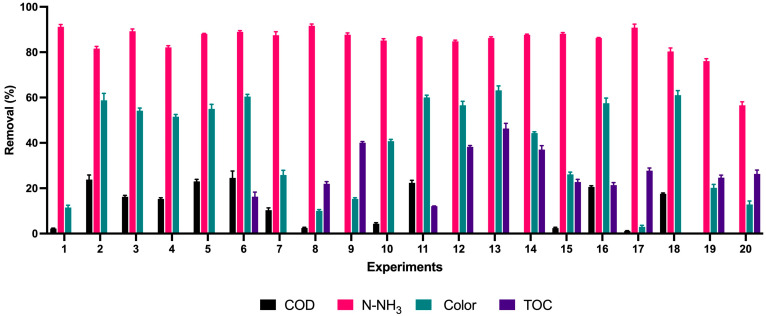
Removal of color, COD, N-NH_3_, and TOC by AOPs with H_2_O_2_/HCO_3_.

Figure 1 shows the changes in color removal in each treatment evaluated. Figure 2 shows the removal efficiencies achieved in each treatment for each parameter assessed.

**Figure 3:**
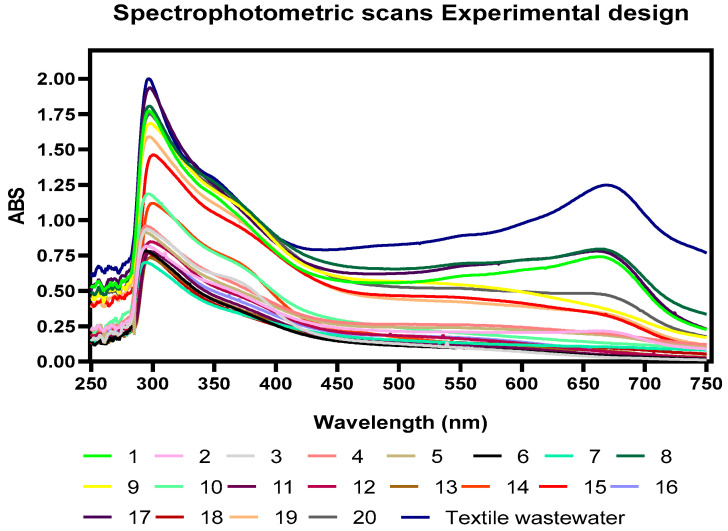
Spectrophotometric scans of wastewater samples from dyeing after treatment with AOPs using HCO_3_^−^/H_2_O_2_ as a catalyst.

According to the spectrophotometric scanning of the wastewater sample, it was found that the highest absorbance values were found at 297 nm and 673 nm. For color removal, experiments 11, 13, 6, 18, 16, and 2 had the highest removal percentages reaching values of 61.05%, 63.15%, 61.4%, 61.05%, 58.8%, 59.45%, respectively, as shown in Figure 2 and visually evidenced in Figure 1. Likewise, the color reductions were not only at the wavelength of 297 nm, where the highest absorbance peak was present (Figure 3); the scans made from a range of 250 nm to 750 nm show significant color reductions in the wavelength of 673 where treatments 11, 13, 6, 18, 16, 2 achieved the removal of up to 98%.

Previous studies have indicated that residual organic carbon in dyehouse wastewater is mainly related to refractory organic compounds, unlike COD which is related to organically bound and inorganic constituents [36,37]; Likewise, it has been reported that TOC is independent of the oxidation state of organic matter and only measures organic carbon converted to CO_2_ [38], suggesting that the observed changes in COD-TOC removal may be related to the degree of changes in the structure of organic compounds after oxidation, which given the complexity of the wastewater matrix generates different intermediary compounds during the oxidation process [39]; this may explain the differences in COD and TOC removals presented in this study.

Some authors have reported by spectrophotometric analysis that peaks at wavelengths between 240 nm–488 nm are characteristic of the Acid Orange (AO 8) dye; Likewise, it has been reported that the absorbance curves in the visible region at 488 nm are associated with the extended chromophore composed of aromatic groups connected through azo bonds, on the other hand, the absorption peaks at 310 and 229 nm in the ultraviolet region are due to the naphthalene ring and benzene ring structures in the dye molecule; it is evident from Figure 1 that the decrease of the observances in these regions indicates the destruction of the azo bonds and benzene rings in the dye present in these regions of the spectrum [40].

It has been reported the degradation of methylene orange (MO) and methylene blue (MB) using HCO_3_^−^/H_2_O_2_ as a catalyst, finding that, for MO, the main absorption spectrum at 464 nm decreases after 30 min, and a new band appears at 251 nm, which would indicate the formation of intermediate products for MB. Authors reported that the absorption bands were present for the wavelengths of 664, 298, and 245 nm and decreased until disappearing after 1 h of treatment. In this case, as evidenced in Figure 3, absorbance peaks similar to those reported for MB were found, which allows intuiting that this is the main dye present in the evaluated effluents [41]. The BAP system is a method that takes advantage of natural activators to increase the profitability of H_2_O_2_-based AOPs; this reaction system, in comparison with other AOPs, is recognized for generating many more ROSs (Reactive Oxygen Species), including peroxymonocarbonate (${\mathrm{HCO}}_{4}^{-}$), superoxide ion ($O_{2}^{.-}$), singlet oxygen (^1^O_2_), hydroxyl radical (OH), and carbonate radical (${\mathrm{CO}}_{3}^{.-}$), which is substrate-dependent (Equations (1)–(6)); this feature has allowed this system to be taken as a successful technology for the oxidative transformation of organic compounds such as azo dyes [42], aliphatic amines [43], aryl sulfide [44,45], and chlorophenols [36,39,41]. Similar results were found in this work. It should be noted that the previously described works were with synthetic wastewater. In contrast, in this work, real dyeing wastewater was used, evidencing the degradation of azo compounds or aromatic groups that could be present in the wastewater. treated with NaHCO_3_ and H_2_O_2_.

(1) $${\mathrm{HCO}}_{3}^{-}+{\;H}_{2}O_{2}\to {\mathrm{HCO}}_{4}^{-}+H_{2}O$$

(2) $${\mathrm{HCO}}_{4}^{-}\to \;\cdot \;{\mathrm{CO}}_{3}^{-}+\cdot \mathrm{OH}$$

(3) $$\cdot \;\mathrm{OH}+{\;\mathrm{HCO}}_{3}^{-}\to \;\cdot \;{\mathrm{CO}}_{3}^{-}+H_{2}O$$

(4) $$H_{2}O_{2}+\;\cdot \;{\mathrm{CO}}_{3}^{-}\;\to {\mathrm{HCO}}_{3}^{-}+{\mathrm{HO}}_{2}$$

(5) $${\mathrm{HO}}_{2}^{\cdot }\to H^{+}+\;\cdot \;O_{2}^{-}$$

(6) $$\cdot \;O_{2}^{-}+\;\cdot \;\mathrm{OH}\to {\mathrm{OH}}^{-}+{\;}^{1}O_{2}^{-}$$

The data obtained from the experimental procedure were studied by Pareto analysis and Surface Response Methodology using STATISTICA 7.0 Software.

**Figure 4:**
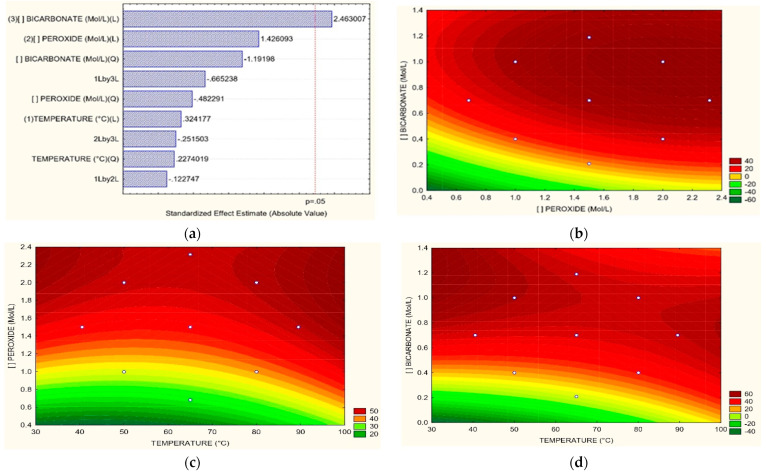
(**a**) Pareto graphic and Surface Response graphics for color removal percentage of (**b**) H_2_O_2_ vs. HCO_3_, (**c**) T vs. H_2_O_2_, (**d**) T vs. HCO_3_.

Figure 4a, corresponding to the Pareto graph, shows that the most influential variable in color removal was the concentration of HCO_3_. Related to H_2_O_2_ vs. HCO_3_, it was observed that it is bicarbonate at concentrations greater than 0.4 M that exerts a significant effect either at low or high concentrations of H_2_O_2_, as shown in Figure 4b. In the case of the relation between T vs. H_2_O_2_, the peroxide exerts a bleaching effect on wastewater from dyeing independent of temperature, at least from concentrations of 1.4 M where temperature can have significant effects at 30 °C and 80 °C as can be seen in Figure 4c, for obtaining removals of 40 to 50% respectively. Concerning T vs. HCO_3_, it is HCO_3_ that has a favorable effect on the removal of dyes over the temperature; from a concentration above 0.4 M of HCO_3,_ the temperature becomes a determining factor in color removal for efficiencies greater than 40%. However, its effect is not very different in ranges from 40 °C to 80 °C if the concentrations of HCO_3_ are high, according to Figure 4d. Other investigations have achieved degradation of the reactive brilliant red dye X-3B in synthetic wastewater using a cobalt(II)/bicarbonate complex (HCO_3_^−^/H_2_O_2_ at 30% *w*/*w*, with concentrations of Co^2+^ of 5 μmol L^−1^, HCO_3_^−^ 10 mmol L^−1^, X-3B 67.5 μmol L^−1,^ and H_2_O_2_ 4 mmol L^−1^); where a pH of 8.2 did not achieve good color removal results using only ions Co^2 +^ in the absence of HCO_3_ when using only low concentrations of HCO_3_^−^ at ten mmol L^−1^ the ratio of degradation was slow; nevertheless, the 85% of the dye was eliminated. When simple cobalt salt was added to the HCO_3_ solution, the discoloration rate increased, corresponding to a positive effect of bicarbonate on color removal, as occurred in this investigation [46]. Zhao et al. [47] reported using H_2_O_2_ and HCO_3_ under low-frequency ultrasonic irradiation to degrade acid orange dye 8 (AO 8). It studied the effect of H_2_O_2_/CO_3_ and ultrasonic irradiation on the AO 8 removal. It reached removals of 3.50%, 6.06%, 15.90%, 65.30%, and 90% when using ultrasound, H_2_O_2_/ultrasound, CO_3_/ultrasound, H_2_O_2_/CO_3_, and H_2_O_2_/CO_3_/ultrasound respectively. It was evidenced that the CO_3_ radical played a significant role in the activated discoloration by ultrasound; again, bicarbonate denoted a positive effect on removals as in the case of our research. This behavior was attributed to the fact that direct pyrolytic fragmentation of AO 8 within bubbles and the supercritical phase reaction at the gas-liquid interface do not dominate its decolorization.

HCO_3_^−^ activated H_2_O_2_ system has many advantages for wastewater treatment, including simple operation, no secondary pollution, strong oxidation capacity, and no reaction selectivity. Regarding the influence of temperature on color removal, there are few reported works; Li et al. [24] carried out experiments at room temperature for the degradation of Acid Orange 7 (AO7) dye at 90 rpm, finding efficiencies of degradation of 33.92 ± 0.86% in 3 h at conditions 0.1 M H_2_O_2_, 0.5 M HCO_3_ and pH 8.6. Kan et al. [20] evaluated the elimination of AO7 at different temperature values, using an initial concentration of AO7 of 5 mg L^−1^, H_2_O_2_ dose of 0.004 mol L^−1^, and HCO_3_ dose of 0.007 mol L^−1^. When the reaction temperature was 298 K, the removal efficiency of AO7 in the H_2_O_2_ system was only 9% in a reaction time of 60 min; the removal rate increased proportionally with temperature, reaching 12% at 328 K. However, the AO7 removal efficiency increased when bicarbonate was added to the process, reaching 30% removal at 298 K and 90% at 398 K in 30 min. The results of this work, like those reported by Kan et al. [20], demonstrated that at high temperatures, reactions occur quickly because the reactants can favorably overcome the energy barrier of the reaction. Similarly, it has been reported that the increase in the reaction temperature favors the decomplexization of Cu-EDTA, which leads to the removal of the color [23].

**Figure 5:**
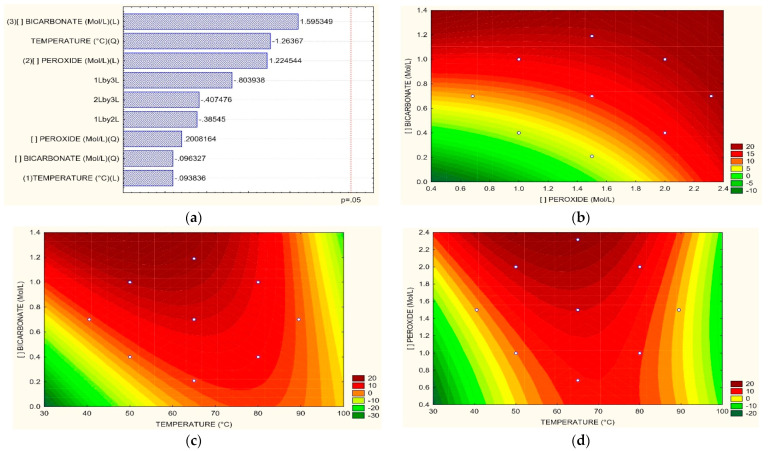
(**a**) Pareto graphic and Surface Response graphics for COD removal percentage of (**b**) H_2_O_2_ vs. HCO_3_, (**c**) T vs. H_2_O_2_, (**d**) T vs. HCO_3_.

Under the evaluated conditions in Table 2, the COD removal percentages were 27.2%, 25.84%, and 23.08%, as shown in Figure 2. As seen in the Pareto graph (Figure 5a), bicarbonate is the most influential variable for COD removal; however, it is not significant enough. The analysis of the surface response concerning H_2_O_2_ vs. HCO_3_ for COD removal shows that the best results are achieved for high concentrations of both H_2_O_2_ and HCO_3_ in the order of 2 M and 1.2 M, respectively; at low concentrations, the effects of COD removal are not efficient as shown in Figure 5b. When the T vs. HCO_3_ relation is evaluated, the high concentration of bicarbonate enhances the COD removal, and temperatures between 70 and 80 °C favor excellent results at different bicarbonate concentration levels, as shown in Figure 5c. Concerning the relation between T vs. H_2_O_2_, the high temperatures improve the reduction of COD, regardless of peroxide concentration; on the other hand, high concentrations of peroxide are meaningful for the removal of COD up to 20% after 1.4 M of H_2_O_2_ according to Figure 5d. Kan et al. [20] reported 72% of COD removal after 60 min of oxidation treatment of synthetic wastewater with Acid Orange II (AO II) dye, using an HCO_3_^−^/H_2_O_2_ system, 180 rpm. It was reported that high temperatures increased the reaction speed, achieving 98.5% of degradation at 54.85 °C. The reaction rate augmented from 0.014 min^−1^ to 0.106 min^−1^ as the reaction temperature increased from 24.85 °C to 54.85 °C using activated H_2_O_2_ with bicarbonate. Thus, temperature proves to be a determining factor in COD removal, as the results obtained in our research. In addition, it has been reported that the efficiency of COD removal is linked to the pH values; the processes with bicarbonate and peroxide maintain a pH between 8.5 and 9 due to the buffering action of the carbonate/bicarbonate [48,49] behavior very similar to the results obtained in the present investigation where the final pH was maintained at values between 8.5 and 9 after adding the bicarbonate; it has been indicated that at low pH with a high concentration of H_2_SO_4_ in the wastewater, it is possible to remove the hydroxyl radical by sulfate, thus reducing the degradation efficiency [49,50]. Finally, the structure of the dye is also an essential factor; cationic dyes by adsorption have a high removal efficiency at high pH, while the negatively charged anionic ones present a better efficiency of COD elimination at neutral or weakly acidic pH; it has been reported that the use of bicarbonate can be inefficient in the elimination of COD bound to anionic dyes because the pH value changes the effect of carbonate and bicarbonate anions. After all, some anions are radical scavengers in the pH range of 8–8.6 [51].

**Figure 6:**
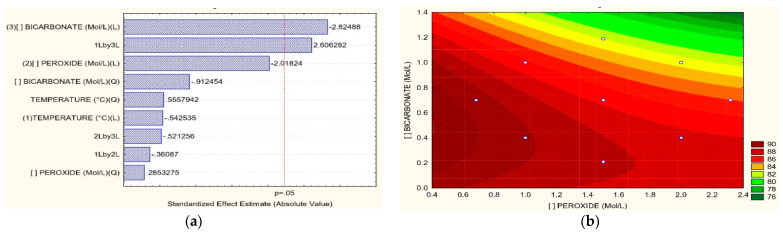

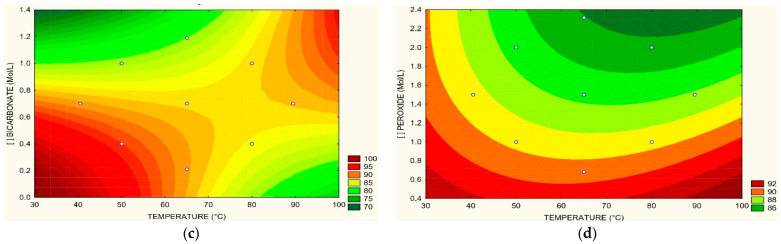
(**a**) Pareto graphic and Surface Response graphics for N-NH_3_ removal percentage of (**b**) H_2_O_2_ vs. HCO_3_, (**c**) T vs. H_2_O_2_, (**d**) T vs. HCO_3_.

For the removal of N-NH_3_, it is observed that in almost all the experiments, it was 80–90%; the best removal efficiencies were 91.15%, 91.73%, and 90.44%, respectively, for experiments 1, 8, and 7, according to Figure 2c. From the Pareto diagram for N-NH_3_ removal (Figure 6a), the concentration of HCO_3_ is the most influent variable over the process; regarding the response surface graphs for the H_2_O_2_ vs. HCO_3_ ratio, at low concentrations, both variables have a significant effect, at concentrations of 0.4 M of bicarbonate and 0.6 M of peroxide (Figure 6b). The relation between T vs. HCO_3_ revealed that the temperature between 30 °C and 40 °C at low concentrations of HCO_3_, like 0.3 M, is efficient, as shown in Figure 6c. In the case of the relation among T vs. H_2_O_2_, temperatures between 30 °C and 40 °C at low concentration of H_2_O_2_, such as 0.6 M, exhibits the best conditions for the removal of N-NH_3_; however, after high values of peroxide at a temperature of 80 °C, good conditions are also presented for removals greater than 85%, as can be seen in Figure 6d.

There is no evidence in the literature regarding the influence of the concentration of HCO_3_ in the elimination of N-NH_3_. Nevertheless, Ruffino and Zanetti [52] reported the removal of N-NH_3_ by the bromide-enhanced ozonation process (BEO), using NaHCO_3_ as a buffer. It was indicated that bicarbonate hinders the acidification of the environment by the action of N-NH_3_, interfering with the formation of nitrates and bromates by capturing OH ions. On the other hand, direct and indirect oxidation transforms organic matter into simpler forms, such as N-NH_3_ or nitrite and nitrate during AOPs, due to the oxidation of the azo groups of the dyes. A study by photo-Fenton found, after 5 min of reaction, nitrification processes with the formation of N-NH_3_ of 2 mg L^−1^ at 15 min and later nitrate formation at 20 min due to the attack of highly oxidizing radicals in textile wastewater [53]. Regarding the influence of H_2_O_2_ on the removal of N-NH_3_, the elimination of N-NH_3_ from 39.0 to 81.2% has been reported using H_2_O_2_ in the treatment of domestic wastewater, so the application of H_2_O_2_ gave rise to the optimization of the nitrification process and elimination of N-NH_3_ thanks to the release of oxygen radicals, which could explain the good results for wastewater from dyeing in our study [54].

**Figure 7:**
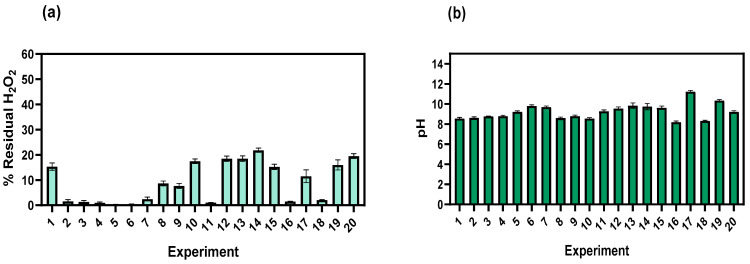
(**a**) Residual H_2_O_2_ percentage in each experiment. (**b**) Final pH in each experiment.

Figure 7a,b show the percentage of residual peroxide and the final pH in each of the experiments; it is evident that the experiments with the highest pollutant load removal in terms of COD and NH_4_ consumed all the peroxide, on average, up to 80% of the peroxide was consumed in the remaining experiments. Hydrogen peroxide plays a crucial role in deciding the pollutant load removal efficiency; a high dose could have the opposite effect and increase the concentration of COD and BOD in the wastewater [55]; H_2_O_2_ can interfere with COD measurements, increase toxicity and decrease the biodegradability of the sample masking the actual behavior of the treated sample; authors have reported that wastewater constituents include a wide variety of organic (carbohydrates, proteins, etc.) and inorganic (phenols, carbonate, sulfates, nitrite, sulfides, chloride) compounds, which react with H_2_O_2_ either competing with organic pollutants for oxidation or forming respective radicals with lower oxidation potential [56,57]. Finally, some components of dyeing wastewater may favor the efficacy of certain AOPs. Figure 7b shows the final pH of the process; all treatments started with sample pH 6.1; it is evident that most treatments were in the range of 8.3–8.8 being 8.2, the lowest value (T16) and 11.3 (T17). Authors have reported that some amounts of HCO_3_^−^ can decompose into CO_2_ or CO_3_ ^2−^ in acidic conditions <4.5 or in strongly alkaline conditions > 11, which could decrease the amount of H_2_O_2_ activating factor [49,58], hence it is possible to obtain a higher decolorization yield in a low alkaline condition (8–9) in an oxidative HCO_3_^−^-H_2_O_2_ process [59]. Similarly, it has been indicated that common pH values in the HCO_3_^−^-H_2_O_2_ system promote the formation of HCO_4_ but do not lead to the formation of ∙OH_2_, and high pH values promote the generation of ∙OH_2_ and HCO_4_^−^; although, if the pH exceeds values above 11 it can affect the presence of H+ and the formation of ^1^O_2_., hence, maintaining a slightly alkaline pH condition allows for an efficient HCO_3_^− _^ H_2_O_2_ system [44].

**Figure 8:**
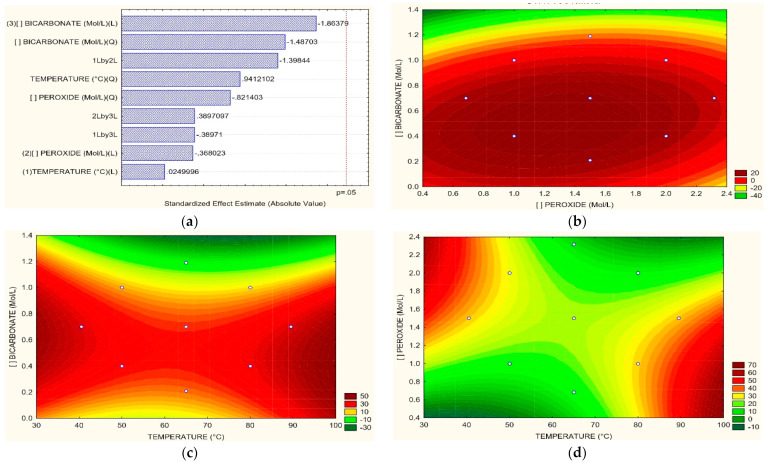
(**a**) Pareto graphic and Surface Response graphics for TOC removal percentage of (**b**) H_2_O_2_ vs. HCO_3_, (**c**) T vs. HCO_3,_ (**d**) T vs. H_2_O_2_.

From the information reported in Figure 2d, it can be seen that experiments 9, 12, and 13 recorded TOC removal percentages of 40.37%, 38.60%, and 47.65%, respectively. The Pareto diagram (Figure 8a) shows no parameter had a significant effect, but the most excellent result corresponds to bicarbonate. In the relation between H_2_O_2_ vs. HCO_3_, shown in Figure 8b, and the connection between H_2_O_2_ vs. HCO_3_, shown in Figure 8b, the optimal conditions for TOC removal are found in the use of 1.4 M of H_2_O_2_ and values of 0.5 of HCO_3_ for an approximate 20% removal. Regarding the relationship T vs. HCO_3_ (Figure 8c), reductions of 50% using 0.6 M HCO_3_ at temperatures relatively close to 30 °C; however, there are good removals at 90 °C using lower amounts of bicarbonate. Finally, the link between T vs. H_2_O_2_ (Figure 8d) exhibits that at high H_2_O_2_ concentrations, among 1.8 and 2.4 M, Good removals of TOC are obtained (around 60% at 30 °C). However, at temperatures over 90 °C would be possible to achieve high removals without the effect of H_2_O_2_ due to the bicarbonate effect over the process. Real textile wastewater with initial TOC values of 60–65 mg L^−1^ and 30 mg L^−1^ were subjected to the electrocoagulation method and adsorption, obtaining removals of 42% where problems have been reported due to the use of excessive amounts of adsorbents that can decrease the performance [60]. Hybrid treatments could also be a good option for TOC elimination since hydrodynamic cavitation has been combined with Fenton’s reagent, obtaining removals of TOC, COD 48%, and 38% of TOC and COD, respectively [61]. Nonetheless, the cost of this process should be reviewed since conventional AOPs with other equipment are usually expensive [19]. When using electrocoagulation, 54% of the removal of TOC in real wastewater was obtained at optimal operating conditions: current density of 10 mA cm^−2^, pH 7 for 10 min. Photo-Fenton process was applied at pH 4.3, Fe_2_^+^ at a concentration of 1.1 mM, and H_2_O_2_ at 9.7 mM during 60 min and 100 rpm; the electrocoagulation + photo-Fenton process achieved 72% of TOC removal [62]. Nanoparticles have also removed TOC in real and synthetic textile wastewater. To degrade Reactive Orange 107 (RO107), Sono-Fenton dye was used with magnetite nanoparticles (Fe_3_O_4_); the optimal conditions for complete decolorization of RO107 were 0.8 g L^−1^ of nanoparticles, pH 5, 10 mM of H_2_O_2_, and 300 W of ultrasonic power reaching removals of 87 and 66.54%, for synthetic and real wastewater respectively [63]. The increase of H_2_O_2_ dosage from 50 to 400 μM in the removal of metronidazole exhibited a positive and significative effect over the speed of the process [64].

### 3.3. Optimum Process Conditions

As a result of the statistical analysis, the respective equations were obtained to determine the optimal conditions for the statistical analysis. In each equation presented in Table 4, T corresponds to the temperature (°C), P to the H_2_O_2_ concentration (Molar), B to the HCO_3_ concentration (Molar), and Z to the removal efficiency (%).

**Figure 9:**
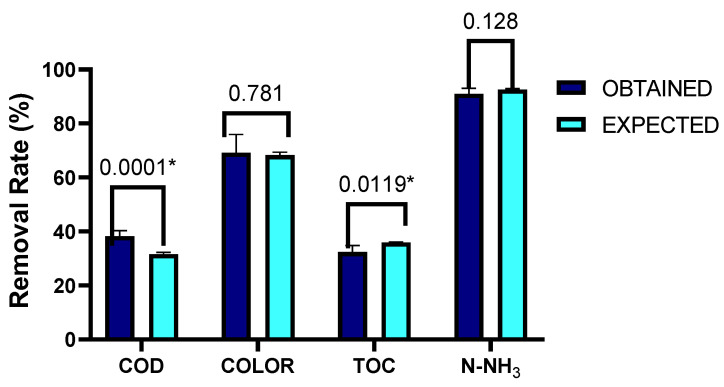
Results of the validation between the expected value yielded by the experimental design and the value obtained experimentally, with *n* = 6. * Significant differences.

From Equations (1)–(12), the optimum conditions were calculated for achieving the highest removal efficiencies. These conditions were 1 M of HCO_3_, 2 M of H_2_O_2_ at 35%, and 60 °C of temperature. Figure 9 shows the validation results between the expected value yielded by the experimental design and those obtained experimentally using six repetitions. To determine statistical significance, multiple t-tests were used for data 1, using the Holm Sidak method, with alpha = 0.05. Each row was analyzed individually without assuming a consistent SD number of *t*-tests: 6. It was found that there are significant differences between what was obtained experimentally and what was expected for the TOC and COD tests. However, it was found that the value was higher in what was obtained experimentally compared to what was expected. This made it possible to establish that the operating conditions obtained were optimal to get the highest removal values for each parameter evaluated.

Then, the AOP treatment was done at these conditions for 1 L of dyeing wastewater at 700 rpm. The results of removal percentages are shown in Table 5 for each parameter evaluated.

Table 5 shows that the optimization increased COD removal up to 31.95% in a time of 60 min; the most significant reduction occurred in the first 20 min of the reaction, as shown in Figure 8; after this time, COD does not vary significantly. It is fundamental to indicate that there are still no reports of COD removal in real wastewater from dyeing using bicarbonate processes with peroxide; there are reports from other AOPs, where the authors have implemented UV/chlorine systems to remove COD in this type of effluents reaching removal percentages in the range of 24.2–29.3% [49]. Kiani et al. [65] reported using peroxydisulfate/CuO and activated carbon under optimum pH seven conditions, achieving 72.1% COD removal percentages in wastewater from dyeing. However, there was a high performance with the use of sulfate radical-based POPs; the side effects in their application are unknown. Some authors have reported essential aspects that affect COD removal percentages and COD analytics in processes with H_2_O_2_ as an oxidizing agent; if removal percentages are low, there may be excess H_2_O_2_, generating residues that do not react in the effluent, which increases the COD concentration of the wastewater [66]. Likewise, inorganic species with reducing capacity, such as nitrite, chloride and H_2_O_2_, can interfere with COD analysis [67].

**Figure 10:**
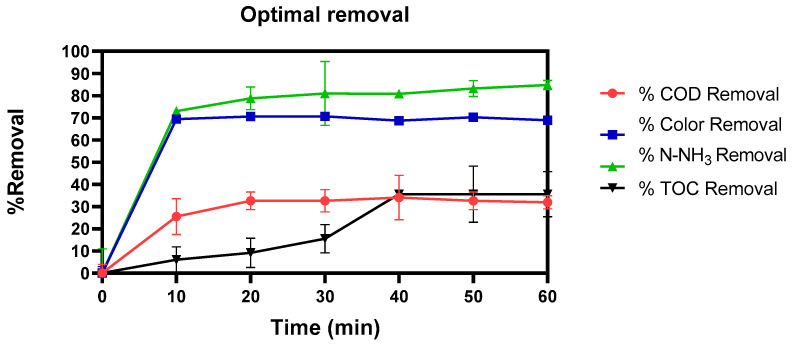
Kinetic of the removal percentage of COD, Color, N-NH_3,_ and TOC at 1 M of HCO_3_, 2 M of H_2_O_2_ at 35%, and 60 °C of temperature.

Figure 10 shows the removal kinetics of the optimal H_2_O_2_ and bicarbonate process, where the behavior of the COD, TOC, color, and N-NH_3_ parameters was analyzed every 10 min.

**Figure 11:**
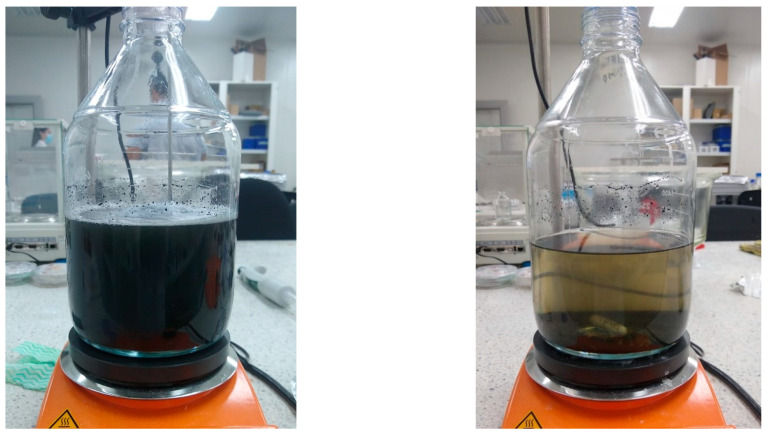
(**left**) The coloration of residual water from dyeing without treatment, (**right**) the coloration of residual water from dyeing after treatment (60 min).

Figure 11 shows the catalytic process before and after under optimization conditions.

Most of the dyes used in the textile industry are synthetic and can be classified into azo, anthraquinone, sulfur, phthalocyanine, and triarylmethane groups; according to their chemical structure and their mode of application, can be classified as reactive, direct, dispersed, primary and vat dyeing [68]. Usually, azo dyes are the most common and constitute 70% of the dyes used in textile wastewater; these are formed by one or more azo bonds, connecting aromatic amines, which makes them rich in nitrogen [3]; the preceding explains the considerable concentrations of N-NH_3_ in the wastewater from the tanneries. Several studies have reported using different AOPs to remove N-NH_3_; processes such as Fenton can mineralize contaminants up to 75.8% N-NH_3_ in 60 min [69]. In the same way, an electro-oxidation process has been reported to improve the elimination of N-NH_3_, achieving removal percentages of up to 90% [70]. Finally, Suryawan et al. [71] said the use of ozone in textile wastewater reached elimination percentages of N-NH_3_ of 46% and 62%. The bicarbonate and hydrogen peroxide system evaluated in this work shows better results concerning the oxidation process and removal of N-NH_3_ than the AOPs applied in tanneries water reported in the literature.

The results obtained concerning the removal of TOC show an efficiency of 35.6%, which reaches a steady state around 40 min of process. Different processes have been reported for the removal of TOC in textile wastewater, most of them synthetic, biological processes and membrane filtrations can reach removals of 44% and 80%, respectively, but in high process times of the order of 30 h and five h are necessary, which is attributed to the degradability of the dyes present in these residual effluents [31,35]. This phenomenon can explain the need to increase the reaction time to achieve more excellent removal according to the results of this work. In Percarbonate/Ultrasound/UV processes, TOC removal percentages were achieved from 32% to 48.6% [72]. The removal of the Orange II (OII) dye and TOC has been reported by advanced oxidation processes using reduced graphene oxide (RGO) as catalysts in the presence of UV/Vis/H_2_O_2_ radiation, reaching removal percentages of 97.7% and 87.9%, respectively in 90 min [73]. These results were relevant considering that no studies have evaluated the effect of NaHCO_3_ and H_2_O_2_ together with parameters such as temperature in real textile wastewater since most studies work with synthetic water with dyes [23,24,25,47,74].

**Figure 12:**
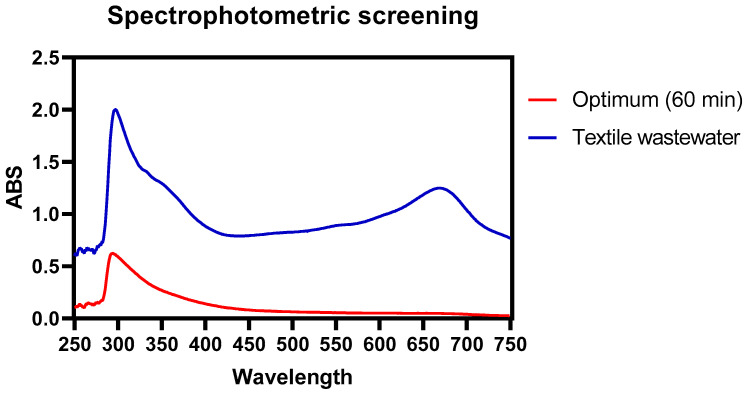
UV-vis absorbance spectra of wastewater samples without treatment and samples treated under optimal conditions (60 min).

The color removals occurred immediately upon adding the reagents to the residual water during the 10 min of reaction, as illustrated in Figure 10, wherein the following 50 min of reaction, the removal percentages are maintained, the absorbances were in a range of 0.55, and 0.62 ABS for 68.85% color reduction. Figure 12 shows the absorbance reduction of the problem sample after treatment, showing the removal of 100% of the color at the 673 nm wavelength and 68.85% at the 297 nm wavelength. An essential factor in removal times is the concentration of the dye in the residual effluents; Some research has highlighted that the removal of methylene blue (MB) and methylene orange (MO) requires more reaction time at higher concentrations of these compounds [41]. One of the characteristics of the dyeing wastewater evaluated in this study is the concentration of chlorides present; it has been reported that chlorides have a dual effect; they can be inhibitors or accelerators in the decolorization kinetic process of dyeing wastewater even in concentrations higher than 5 mM up to 500 Mm [74]; however it has also been indicated that processes such as ozonation [75], and UV/TiO_2_ [26], can be affected in the decolorization rate in this range of chloride concentrations; for the case of this study, the chloride concentration was 14. 55 mM and it was evidenced that color removal was not significantly affected; similar results were reported when NaCl was added to a UV/H_2_O_2_ process, where the removal rate was not significantly affected; the slight decrease in decolorization efficiency is because of hydroxyl radical scavenging of the chloride ion (Equations (19) and (20)) [76].

(19) $${\mathrm{Cl}}^{-}+\;\cdot \;H\to {\;\mathrm{HOCl}}^{-}$$

(20) $${\mathrm{HOCl}}^{-}+{\;H}^{+}\;\to \mathrm{Cl}\;\cdot \;+{\;H}_{2}O$$

**Figure 13:**
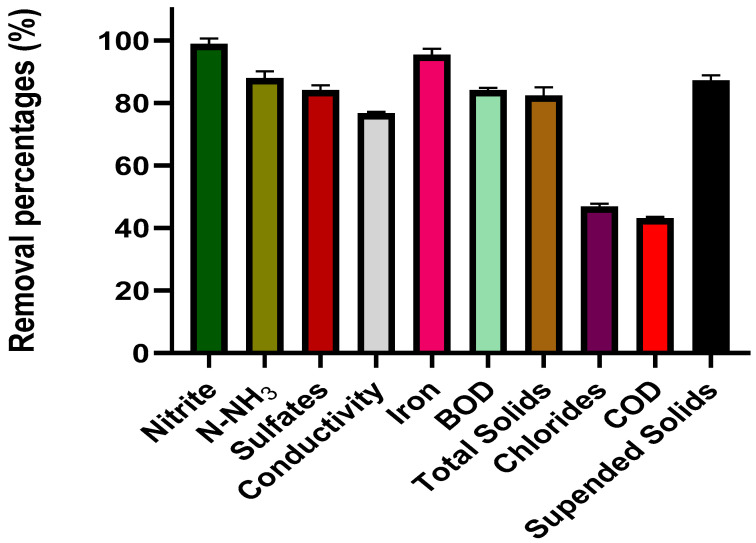
Removal values of nitrite, N-NH_3_, sulfates, conductivity, iron, BOD, total solids, chlorides, COD and suspended solids.

Another critical factor in eliminating dyes in textile waters is pH; it has been estimated that at a pH of 4.5 to 7.3, the bicarbonate ions released interact with the dyes allowing their degradation [77,78]. About the other pollutants, it was found that the removal was above 80% (nitrites, N-NH_3_, sulfates, iron, BOD, total solids, and suspended solids); for the other parameters evaluated in the characterization, the removal was between 42% and 77% as shown in Figure 13; it is essential to note that all the concentration and removal values obtained comply with the current regulations for the discharge of this type of wastewater.

The perspective on the use of the bicarbonate/peroxide system in the treatment of dyehouse effluents lies in the determination of the cost-effectiveness ratio; in the literature, there is little data on the costs associated with hydrogen peroxide activation due to its dependence on the effluent and the characteristics of the process itself [77,78]; however, some works report costs associated with the operation, reagent consumption, and electricity consumption (Table 6).

For the treatment of coloring effluents, it has been reported that ozone alone is more expensive than other processes, such as H_2_O_2_/UV and ozone/UV, due to electrical consumption. Treating these effluents with bicarbonate decreases energy costs by having a lower energy consumption, and the construction of the reactors is usually cheaper than photocatalytic reactors [79]. The use of the Bicarbonate/peroxide complex allows for obtaining some chemically oxidative species such as OH, O_2_, and O_2_-; and therefore, they could be simultaneously used for the decomposition of organic pollutants present in dyeing effluents [80,81], being a potentially cheaper technology than those currently evaluated.

**Table 6:** Costs by year of dye effluent treatment by different AOPs related to operation and reagent and electricity consumption.

AOP	Volume Treated/Year (m^3^)	Labor Costs (€)	Reagent Cost (€)	Energy Costs (€)	Total Cost/Year (€)	Reference
O_3_	63,170	61,957.07	0	483,717.81	545,674.88	[80]
O_3_/UV	90,880			419,403.16	481,360.23	
H_2_O_2_/O_3_/UV	49,600		2338.71	323,855.36	388,151.15	
H_2_O_2_/UV	22,450		3178.01	54,488.27	119,623.36	
Solar/Fe(II)/H_2_O_2_	365,000	12,100	33,400	20,100	65,600	[81]
Solar/Fe(II)/PDS			145,300		177,500	
UV/PDS			27,300	50,500	89,900	
UV/H_2_O_2_			1800	62,100	76,000	

## 4. Conclusions

The Advanced Oxidation Processes using H_2_O_2_ and NaHCO_3_ showed promising results for removing color, N-NH_3_, and to a lesser extent, the reduction of COD and TOC. Even though the reaction temperatures that showed a relative efficiency are between 50 °C and 80 °C, the energy expenditure could be a limitation in a scaled process; nevertheless, thanks to the analysis of the surface response, it can be detailed that by increasing the concentrations of NaHCO_3_ and temperature it can be obtained excellent results. On the other hand, to get high removals of N-NH_3_ after the treatment, concentrations of peroxide and low temperatures are necessary. Therefore, the appropriate type of treatment should be prioritized according to the intended use of the treated effluent since the dyeing water treated with AOPs could be used for the potential cultivation of microalgae, given the high amounts of nitrates that can be generated by oxidation and degradation as a secondary treatment; in this way, the conditions that favor the oxidation of N-NH_3_ should be used; on the other hand, if what is sought is the removal of color and the COD, other conditions should be analyzed, more research is required to evaluate the potential use of the treated water and some other parameters that may favor the treatment of said water.

## Figures and Tables

**Table 1:** Physicochemical characterization of dyeing effluents.

Parameter	Units	Standard Methods Code
COD	mg × L^−1^	5220C
BOD		5210B-4500-OG
Nitrates		4500-NO_3_ B
Nitrites		4500-NO_2_ B
Ammonia nitrogen		4500-NH_3_ F
Phosphates		4500-P C
Total Suspended Solids (mg × L^−1^)		2540D
Heavy Metals Fe, Cr (mg × L^−1^)		3111D
Sulfides (mg × L^−1^)		4500-S_2_ F
Chlorides (mg × L^−1^)		4500-ClB
pH	pH units	4500B
Conductivity	µS × cm^−1^	2510B

**Table 2:** Experimental design conditions.

P	C	T1	T2	T3	T4	T5	T6	T7	T8	T9	T10	T11	T12	T13	T14	T15	T16	T17	T18	T19	T20
T (°C)	28	50	50	50	80	80	65	65	50	50	80	80	65	65	40.5	89.5	65	65	65	65	65
H_2_O_2_ (M)	0	1	1	2	1	2	1.5	1.5	1	2	1	2	1.5	1.5	1.5	1.5	0.68	2.31	1.5	1.5	1.5
HCO_3_ (M)	0	0.4	0.4	1	1	0.4	0.7	0.7	1	0.4	0.4	1	0.7	0.7	0.7	0.7	0.7	0.7	0.21	1.18	0.7

Where P = parameter, C = control and T = treatment.

**Table 3:** Characterization of dyeing wastewater.

Parameters	Unit	This Research	[27]	[28]	[29]	[30]	[31]	[32]	[33]
Nitrate	mg L^−1^	65	9.3	-	29.06 ± 1.67	-	86.87	-	5.18
Nitrite	mg L^−1^	1.25	0.004	-	-		-	-	-
N-NH_3_	mg L^−1^	35.6	-	-	-	10 ± 4.35	-	-	-
Sulfates	mg L^−1^	385	112	275	-	-	629	1384	8.98
Conductivity	µS cm^−1^	1145	2269	5.2	69.0 ± 0.05	-	2836	4010	1675
pH	-	6.1	7.9	7.5	7.63 ± 0.10	6.25 ± 0.16	11.84	7.39	4.34
Iron	mg L^−1^	7.5	0.1	2.35	-	17.77 ± 0.20	-	-	-
Chrome	mg L^−1^	N.D	-	-	-	-	-	-	-
BOD	mg L^−1^	225.56	-	248	9.33 ± 1.03	782 ± 8.02	20	474	2667
Total solids	mg L^−1^	1367	3.6	4722	-	5483 ± 12.58	-	-	-
Chlorides	mg L^−1^	850.67	506	2586	-	286 ± 7	839.58	-	198.39
COD	mg L^−1^	598	65	689	18.44 ± 1.62	1700 ± 11.53	1024.6	1088	12.69
Suspended solids	mg L^−1^	587.43	-	235	-	-	0.011	68	-
Type of water		Real	Real	Real	Real	Real	Real	Real	Real

**Table 4:** Statistical analysis of experimental data for determining optimal conditions.

Relation	Equation	
For Color		
T = 65 °C H_2_O_2_ vs. HCO_3_	$Z=-129.835+65.176P-10.933P^{2}+239.66B-75.056B^{2}-0.119\times 65P-1.076\times 65B-12.208PB+44.328$	(7)
H_2_O_2_ = 1.5 M T vs. HCO_3_	$Z=-129.835+0.309T+0.006T^{2}+239.661B-75.056B^{2}-0.119\times 1.5T-1.076TB-12.208\times 1.5B+73.166$	(8)
HCO_3_ = 0.7 M T vs. H_2_O_2_	$Z=-129.835+0.301T+0.057T^{2}+65.176P-10.933P^{2}-0.119TP-1.076\times 0.7T-12.208\times 0.7P+130.985$	(9)
For COD		
T = 65 °C H_2_O_2_ vs. HCO_3_	$Z=139.912+20.587P+2.445P^{2}+82.027B-3.258B^{2}-0.201\times 65P-0.699\times 65B+10.624PB-122.393$	(10)
H_2_O_2_ = 1.5 M T vs. HCO_3_	$Z=139.912+2.994T-0.017T^{2}+82.027B-3.258B^{2}-0.201\times 1.5T-0.699TB-10.625\times 1.5B+36.382$	(11)
HCO_3_ = 0.7 M T vs. H_2_O_2_	$Z=139.912+2.994T-0.017T^{2}+20.587P+2.445P^{2}-0.201TP-0.699\times 0.7T-10.625\times 0.7P+55.823$	(12)
For N-NH_3_		
T = 65 °C H_2_O_2_ vs. HCO_3_	$Z=118.176-0.535T+0.0017T^{2}-25.115B-6.909B^{2}-0.042\times 1.5T+0.507TB-3.043\times 1.5B+1.446$	(13)
H_2_O_2_ = 1.5 M T vs. HCO_3_	$Z=118.176-0.535T+0.0017T^{2}-0.202P+0.778P^{2}-0.042TP+0.507\times 0.7T-3.043\times 0.7P-20.967$	(14)
HCO_3_ = 0.7 M T vs. H_2_O_2_	$Z=118.176-0.535P+0.0017P^{2}-0.203B+0.7778B^{2}-0.042PB+0.507\times 0.7P-3.043\times 0.7B-20.967$	(15)
For TOC		
T = 65 °C H_2_O_2_ vs. HCO_3_	$Z=-55.937-0.464T+0.0185T^{2}+84.834B-73.103B^{2}-1.0599\times 1.5T-0.492TB+14.769\times 1.5B+115.684$	(16)
H_2_O_2_ = 1.5 M T vs. HCO_3_	$Z=-55.937-0.464T+0.0185T^{2}+98.928P-14.537P^{2}-1.0599TP-0.492\times 0.7T+14.76\times 0.7P+23.563$	(17)
HCO_3_ = 0.7 M T vs. H_2_O_2_	$Z=-55.937+98.928P-14.537P^{2}+84.834B-73.1027B^{2}-1.0599\times 65P-0.492\times 65B+14.69PB+48.025$	(18)

**Table 5:** Mean removal percentages at optimum conditions.

Parameter	% of Removal
N-NH_3_	92.35
COD	31.94
Color	68.85
TOC	35.60

## Data Availability

Not applicable.
